# Supplementary material for: The Evolutionary History of New Zealand Deschampsia Is Marked by Long-Distance Dispersal, Endemism, and Hybridization
Source: Biology (Basel). 2021 Oct 5;10(10):1001. doi: 10.3390/biology10101001 (PMC8533413; doi:10.3390/biology10101001)
Supplement: Supplementary file 1 [file biology-10-01001-s001.zip › Figure S2.pdf]

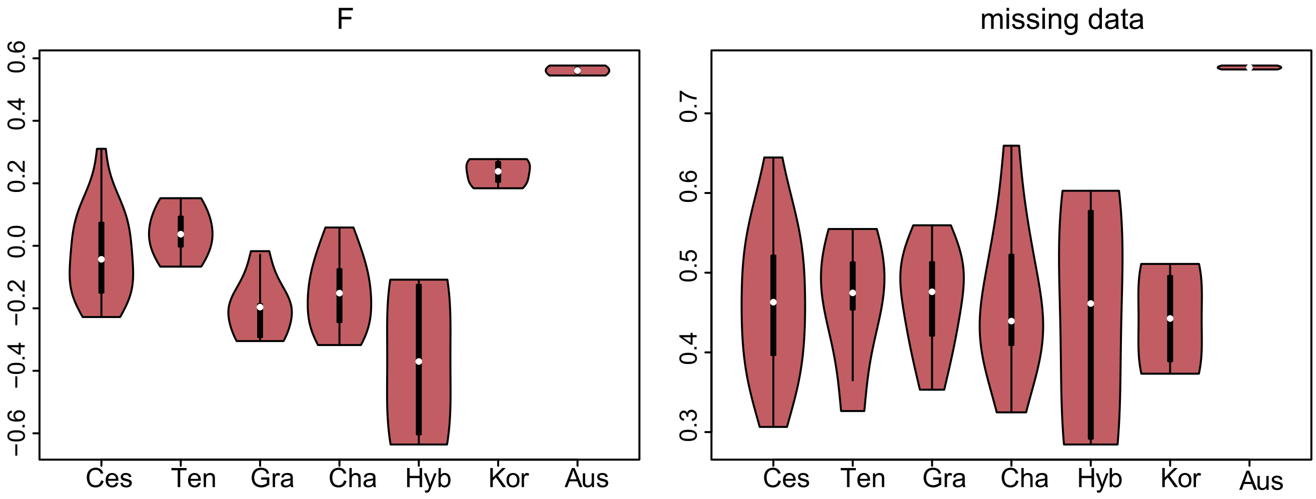

FigureS2. Vioplots of inbreeding coefficient (F) and proportion of missing data estimated by Vcftools for all species in this study. Ces = *D. cespitosa* (NZ), Ten = *D. tenella*, Gra = *D. gracillima*, Cha = *D. chapmanii,* Kor = *D.cespitosa* of Korea, Aus = *D.cespitosa* of Australia, Hyb = Hybrid population.
