# Supplementary material for: The Evolutionary History of New Zealand Deschampsia Is Marked by Long-Distance Dispersal, Endemism, and Hybridization
Source: Biology (Basel). 2021 Oct 5;10(10):1001. doi: 10.3390/biology10101001 (PMC8533413; doi:10.3390/biology10101001)
Supplement: Supplementary file 1 [file biology-10-01001-s001.zip › Figure S3.pdf]

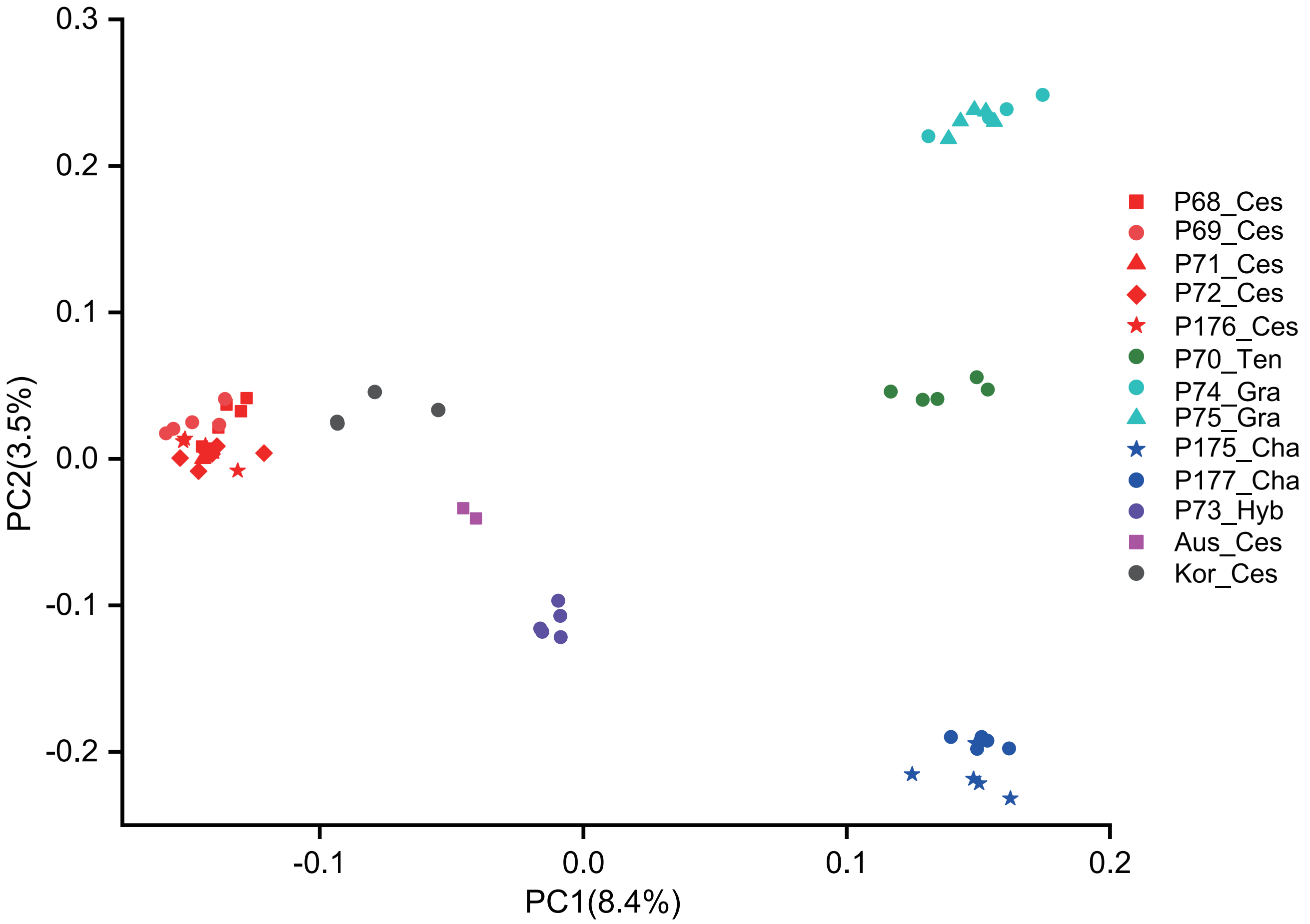

FigureS3. PCA results of 57 accessions sampled across 13 sampling localities based on 622,478 polymorphic sites from PCAngsd results. Each population is represented in different color or shape: Ces = *D. cespitosa*, Ten = *D. tenella*, Gra = *D. gracillima*, Cha = *D. chapmanii*. Hyb = Hybrid population, Aus = Australia polulation, Kor = Korea population.
