# Supplementary material for: The Evolutionary History of New Zealand Deschampsia Is Marked by Long-Distance Dispersal, Endemism, and Hybridization
Source: Biology (Basel). 2021 Oct 5;10(10):1001. doi: 10.3390/biology10101001 (PMC8533413; doi:10.3390/biology10101001)
Supplement: Supplementary file 1 [file biology-10-01001-s001.zip › Figure S7.pdf]

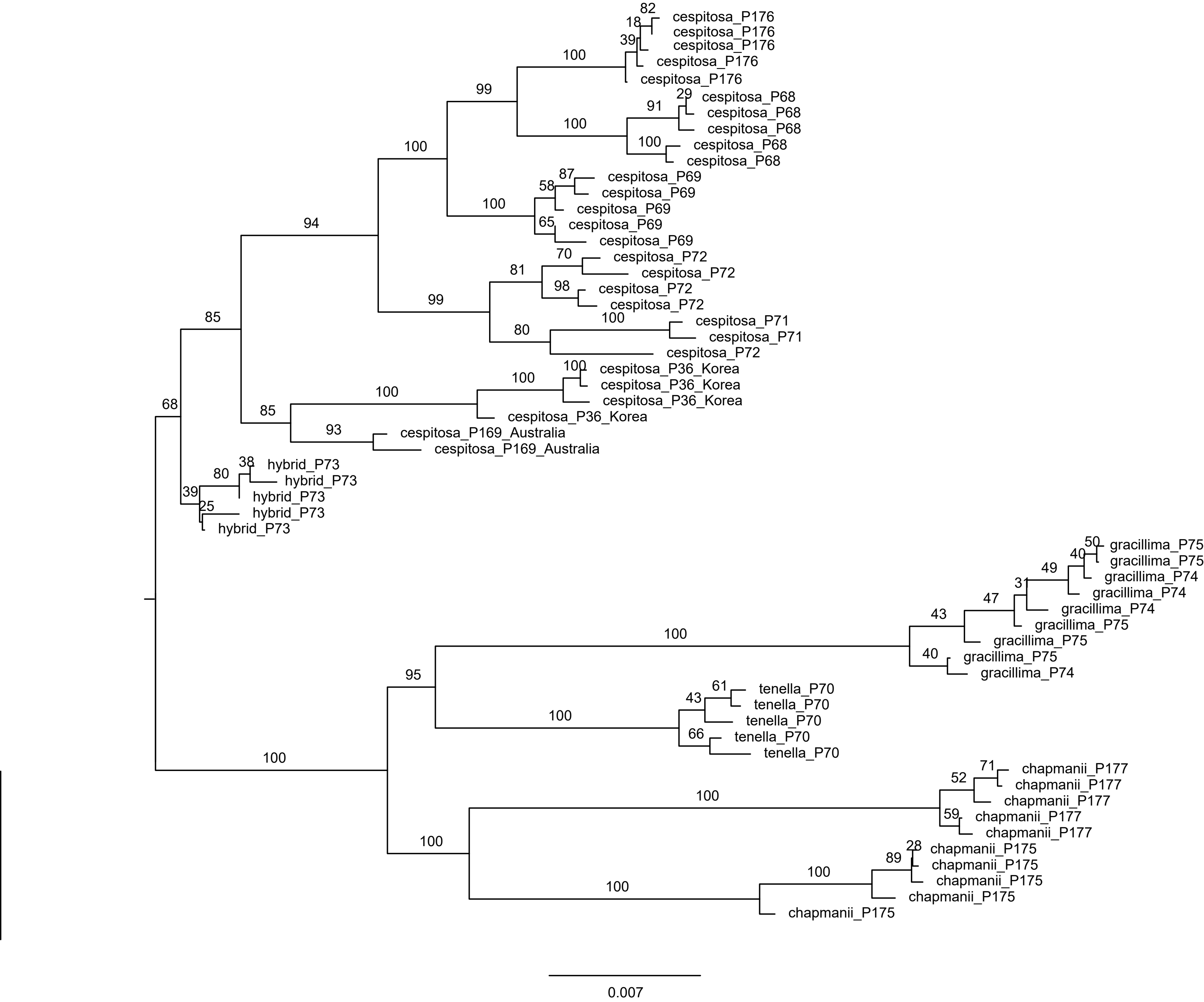

Figure S7. The best scoring maximum likelihood phylogenetic tree derived from 3,252 SNPs from 57 individuals (including hybrid population). The tree was rooted using *Hordeum vulgare* as outgroup (not shown). Bootstrap support is shown on the branches of tree.
