# Supplementary material for: The Evolutionary History of New Zealand Deschampsia Is Marked by Long-Distance Dispersal, Endemism, and Hybridization
Source: Biology (Basel). 2021 Oct 5;10(10):1001. doi: 10.3390/biology10101001 (PMC8533413; doi:10.3390/biology10101001)
Supplement: Supplementary file 1 [file biology-10-01001-s001.zip › Figure S8.pdf]

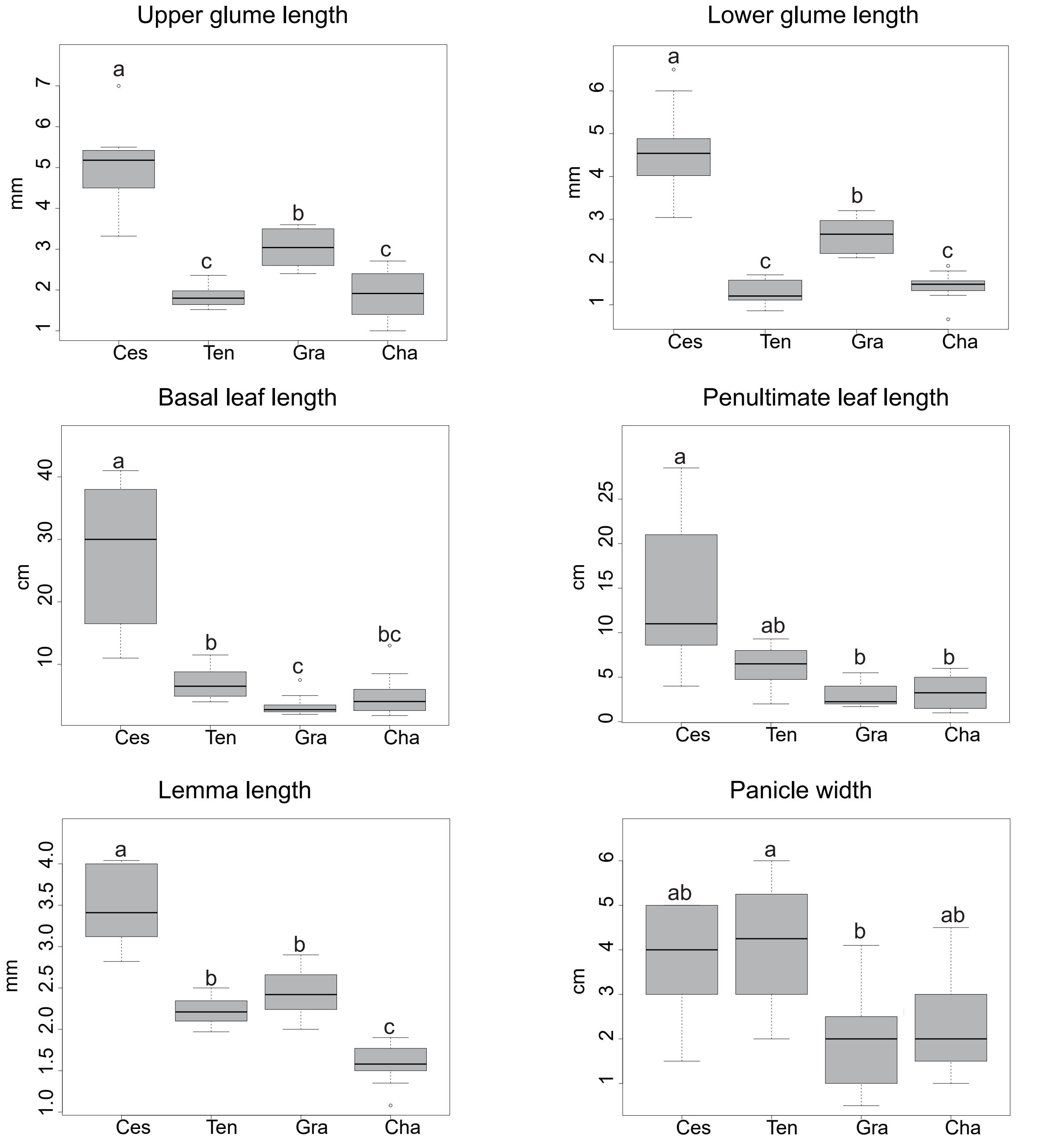

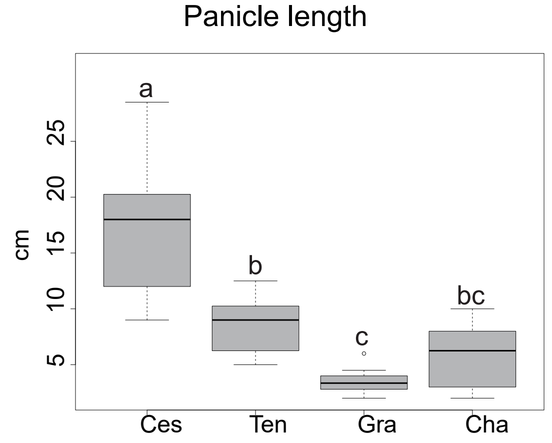

Figure S8. Boxplots showing variation in morphological traits in New Zealand *Deschampsi cespitosa* and three endemic taxa. Levels not connected by the same letter are significantly different according to the Mann-Whitney-U-tests ($p < 0.05$). Taxa codes: Ces = *D cespitosa,* Ten = *D. tenella*, Gra = *D. gracillima*, Cha = *D. chapmanii*. P values of Mann-Whitney-U-tests for pairwise comparisons are shown in Table 1. Additional boxplots are given in Fig. 5
