# Supplementary material for: The Evolutionary History of New Zealand Deschampsia Is Marked by Long-Distance Dispersal, Endemism, and Hybridization
Source: Biology (Basel). 2021 Oct 5;10(10):1001. doi: 10.3390/biology10101001 (PMC8533413; doi:10.3390/biology10101001)
Supplement: Supplementary file 1 [file biology-10-01001-s001.zip › Table S1_resubmission.pdf]

Table S1. Information on our samples used in this study: Pop ID, population accession number; Ind ID, individual number; No. RAD: number of individuals included in RAD analysis; No. Plastid: number of individuals included in plastid analysis; NZ = New Zealand; UK = United Kingdom; ARG = Argentina, EST = Estonia, ISL = Iceland, RUS = Russia.

Pop ID	Species	Country	Latitude	Longitude	No. RAD	No. Plastid
169	*D. cespitosa*	Australia	-54.63	158.82	2	0
172	*D. cespitosa*	Australia	-32.742	145.559	1	1
36	*D. cespitosa*	Korea	33.362	126.518	4	1
68	*D. cespitosa*	NZ	-38.729	177.131	5	0
69	*D. cespitosa*	NZ	-39.361	176.363	5	1
176	*D. cespitisa*	NZ	-43.242	170.130	5	1
71	*D. cespitosa*	NZ	-45.747	167.367	2	1
72	*D. cespitosa*	NZ	-45.747	167.367	5	1
73	*D. ces×chap*	NZ	-45.747	167.367	5	1
175	*D. chapmanii*	NZ	-41.516	172.553	5	0
177	*D. chapmanii*	NZ	-43.498	171.165	5	1
74	*D. gracillima*	NZ	-52.575	169.165	4	1
75	*D. gracillima*	NZ	-52.572	169.169	5	0
70	*D. tenella*	NZ	-45.773	167.369	5	1
Further individuals only in chloroplast genome analysis						
Ind ID	Species	Country	Latitude	Longitude	No. RAD	No. Plastid
812	*D.koelerioides*	China	43.111	86.841	0	1
803	subsp. *pamirica*	China	43.808	87.998	0	1
804	subsp. *pamirica*	China	43.1198	86.989	0	1
820	subsp. *orientalis*	China	37.300	101.420	0	1
863	subsp. *orientalis*	China	29.882	102.019	0	1
1	*D. cespitosa*	Austria	47.426	13.615	0	1
59	*D. cespitosa*	UK	57.607	-4.951	0	1
242	*D. cespitosa*	EST	58.604	22.690	0	1
516	*D. cespitosa*	ISL	63.892	-21.364	0	1

534	*D. cespitosa*	Canada	47.020	-67.729	0	1
620	*D. cespitosa*	RUS	69.218	35.397	0	1
991	*D. cespitosa*	RUS	66.115	170.522	0	1
1012	*D. cespitosa*	RUS	51.195	40.308	0	1
297	*D. cespitosa*	ARG	-44.897	-71.51	0	1
319	*D. cespitosa*	ARG	-41.196	-71.832	0	1
307	*D. antarctica*	ARG	-44.897	-71.51	0	1
