# Supplementary material for: The Evolutionary History of New Zealand Deschampsia Is Marked by Long-Distance Dispersal, Endemism, and Hybridization
Source: Biology (Basel). 2021 Oct 5;10(10):1001. doi: 10.3390/biology10101001 (PMC8533413; doi:10.3390/biology10101001)
Supplement: Supplementary file 1 [file biology-10-01001-s001.zip › Table S2.pdf]

Table S2. The measured morphological characters of *Deschamsia* in New Zealand.

Acronym	Character	Measure/explanation
Hplant	Plant height	cm
Lpanic	Panicle length	cm
Wpanic	Panicle width	cm
Lleaf_pen	Leaf length -penultimate	cm
Lleaf_bas	Leaf length -basal	cm
Sleaf	Leaves shape	0=flat, 1=filiform
Nflorets	Florets per spikelet	n
Lloglume	lower glume length	mm
Lupglume	Upper glume length	mm
Llemma	Lemma length	mm
PRawn	Awn presence	0=absent 1=present
IPawn	Awn insertion point	0=basal third, 1=medial third, 2=upper third or apical
Lawn	Awn length	mm
Sawn	Awn shape	0=straight 1=bent
