# Supplementary material for: The Evolutionary History of New Zealand Deschampsia Is Marked by Long-Distance Dispersal, Endemism, and Hybridization
Source: Biology (Basel). 2021 Oct 5;10(10):1001. doi: 10.3390/biology10101001 (PMC8533413; doi:10.3390/biology10101001)
Supplement: Supplementary file 1 [file biology-10-01001-s001.zip › Table S3.pdf]

Table S3. Bonferroni-corrected P values of Kruskall-Wallis-test between all *Deschampsia* taxa of New Zealand.

Character	All taxa
Plant height	p= 0.000
Panicle length	p= 0.000
Panicle width	p= 0.020
Penultimate leaf length	p= 0.000
Basal leaf length	p= 0.000
Lower glume length	p= 0.000
Upper glume length	p= 0.000
Lemma length	p= 0.000
Awn length	p= 0.049
